# Binocular integration of chromatic and luminance signals

**DOI:** 10.1167/jov.24.12.7

**Published:** 2024-11-05

**Authors:** Daniel H. Baker, Kirralise J. Hansford, Federico G. Segala, Anisa Y. Morsi, Rowan J. Huxley, Joel T. Martin, Maya Rockman, Alex R. Wade

**Affiliations:** 1 Department of Psychology, University of York, York, UK; 2 York Biomedical Research Institute, University of York, York, UK; 3 School of Psychology, University of Nottingham, Nottingham, UK; 4 School of Philosophy, Psychology and Language Sciences, University of Edinburgh, Edinburgh, UK

**Keywords:** *isoluminant*, *dichoptic*, *binocular interactions*, *gain control*, *summation*, *suppression*, *masking*, *psychophysics*

## Abstract

Much progress has been made in understanding how the brain combines signals from the two eyes. However, most of this work has involved achromatic (black and white) stimuli, and it is not clear if the same processes apply in color-sensitive pathways. In our first experiment, we measured contrast discrimination (“dipper”) functions for four key ocular configurations (monocular, binocular, half-binocular, and dichoptic), for achromatic, isoluminant L-M and isoluminant S-(L+M) sine-wave grating stimuli (L: long-, M: medium-, S: short-wavelength). We find a similar pattern of results across stimuli, implying equivalently strong interocular suppression within each pathway. Our second experiment measured dichoptic masking within and between pathways using the method of constant stimuli. Masking was strongest within-pathway and weakest between S-(L+M) and achromatic mechanisms. Finally, we repeated the dipper experiment using temporal luminance modulations, which produced slightly weaker interocular suppression than for spatially modulated stimuli. We interpret our results in the context of a contemporary two-stage model of binocular contrast gain control, implemented here using a hierarchical Bayesian framework. Posterior distributions of the weight of interocular suppression overlapped with a value of 1 for all dipper data sets, and the model captured well the pattern of thresholds from all three experiments.

## Introduction

The process by which the brain combines independent inputs is of fundamental importance for understanding sensory perception (Baker & Wade, 2017; Ernst & Banks, 2002). Binocular vision is a useful test case for determining the general principles involved in neural signal combination, as our brains typically combine the inputs from the left and right eyes to provide binocular single vision (Read, 2021). In recent years, our understanding has been facilitated by the development of binocular gain control models that provide a framework to interpret empirical data from multiple experimental paradigms and techniques, including psychophysics (Meese, Georgeson, & Baker, 2006), EEG (Baker & Wade, 2017), fMRI (Moradi & Heeger, 2009), and pupillometry (Segala et al., 2023). However, most of this work has used achromatic (black and white) stimuli; we know comparatively little about how chromatic signals are combined binocularly or how signals in different ocular and chromatic channels interact. In this study, we use psychophysical detection and discrimination paradigms to explore binocular interactions within and between the chromatic and achromatic pathways.

**Figure 1.**
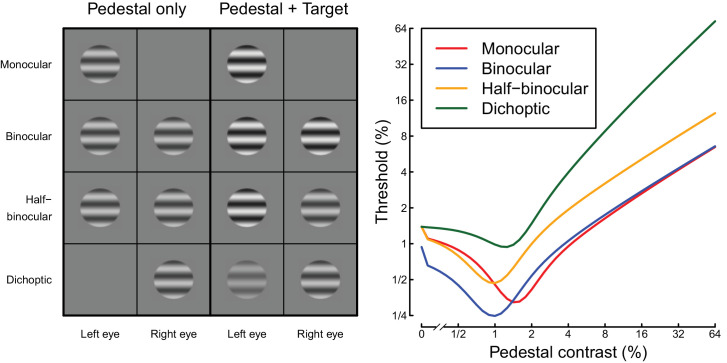
Illustration of stimulus conditions (left) and example dipper functions (right).

A useful framework for understanding binocular signal processing is the two-stage gain control model of binocular combination introduced by Meese, Georgeson, and Baker (2006). This model features interocular suppression between monocular channels, followed by binocular summation. The model accounts well for the pattern of contrast discrimination (“dipper”) functions for four distinct ocular configurations (see also Georgeson, Wallis, Meese, & Baker, 2016), illustrated in Figure 1. In the monocular condition, participants must discriminate between stimuli of two contrasts (a “pedestal” and a “pedestal plus target”) that are both presented to one eye, while the other eye views mean luminance. Threshold is defined as the minimum target contrast required to make this judgment with 75% accuracy; this reduces at pedestal contrasts around threshold (facilitation) and increases at high pedestal contrasts (masking). The binocular condition is the same, except that the stimuli are shown to both eyes. In the half-binocular condition, the pedestal is shown to both eyes, but the target increment is shown only to one eye. Finally, the dichoptic condition involves presenting the pedestal to one eye and the target increment to the other eye.

The detailed pattern of thresholds across these four conditions is complex and for achromatic stimuli has several distinctive features that have been replicated in multiple studies. At low pedestal contrasts, the binocular condition yields lower thresholds than the monocular condition; this result is attributed to physiological binocular summation by neurons responsive to signals from both eyes (Baker, Lygo, Meese, & Georgeson, 2018; Campbell & Green, 1965). However, at high pedestal contrasts, the “handle” regions of the dipper functions for these conditions converge: a consequence of interocular suppression compensating for the increased excitation during binocular stimulation (Legge, 1984; Maehara & Goryo, 2005). The half-binocular condition avoids confounding the number of eyes seeing the target with the number of eyes seeing the pedestal (Meese, Georgeson, & Baker, 2006). The pedestal is always binocular in this condition, whereas the target increment is monocular, and thresholds are consistently higher than in the binocular condition across the full range of pedestal contrasts. This demonstrates that binocular summation occurs across the entire contrast range, when the pedestal ocularity is appropriately controlled. Finally, the dichoptic condition produces extremely strong masking of the target, such that when the pedestal is visible, the target must equal or exceed its contrast to be detectable (Baker & Meese, 2007; Legge, 1979; Maehara & Goryo, 2005). The characteristic pattern of dipper functions (right panel of Figure 1) is well-described by the gain control model of Meese, Georgeson, and Baker (2006) for achromatic stimuli.

At the output of the human retina, cone responses are split into three distinct pathways. The sum of long-wavelength (L) and medium-wavelength (M) cone outputs (L+M) transmits luminance information (see Sharpe, Stockman, Jagla, & Jägle, 2011) and is likely responsible for the binocular combination effects previously studied using achromatic stimuli (see above). The difference of long- and medium-wavelength cone outputs (L-M) is responsive to chromatic stimuli modulating along a red/green axis in color space. Finally, the opponent short-wavelength (S) channel codes chromatic stimuli modulating along a blue/yellow axis (S-(L+M)). At isoluminance, where (L+M) is held constant, this channel is driven purely by the S-cone outputs. Note that we use these shorthand terms throughout to refer to chromatic mechanisms but acknowledge that in reality, the photoreceptor outputs are weighted, for example, S-0.5(L+M) and 3L+M. Within each chromatic pathway, contrast discrimination functions (which plot discrimination thresholds against pedestal contrast; sometimes referred to as TvC functions) are similar to those for achromatic vision, showing evidence of facilitation at low pedestal contrasts and masking at high pedestal contrasts (Chen, Foley, & Brainard, 2000; Switkes, Bradley, & De Valois, 1988). There are also interactions between luminance and color mechanisms, which are able to mask each other (Chen et al., 2000; Mullen & Losada, 1994; Switkes et al., 1988), and under some circumstances also show cross-pathway facilitation (Cole, Stromeyer, & Kronauer, 1990; Mullen & Losada, 1994; Shooner & Mullen, 2020; Switkes et al., 1988).

There has not yet been a detailed investigation of binocular contrast interactions in either chromatic pathway, but there are reasons to believe they may differ from the achromatic pathway. At detection threshold, binocular summation is greater for chromatic versus achromatic stimuli (Simmons, 2005), implying a more linear initial stage of processing. For cross-orientation masking, there are differences in the magnitude of masking between chromatic (L-M) and achromatic stimuli (Kim, Gheiratmand, & Mullen, 2013; Medina & Mullen, 2009), as well as differences in their temporal dynamics (Kim & Mullen, 2015). There are also binocular interactions between chromatic and achromatic pathways (Kingdom & Libenson, 2015; Mullen, Kim, & Gheiratmand, 2014), yet these have not been fully explored for arrangements where the target and mask have the same orientation. Finally, the neurophysiological underpinnings of color vision may be distinct from those of the achromatic system. The classical view was that in primary visual cortex (V1), chromatic signals are processed in “blob” regions that are revealed by cytochrome oxidase staining (Horton & Hubel, 1981) and are largely monocular (Livingstone & Hubel, 1984). However, subsequent work has revealed a large population of color-luminance neurons with a subset of color-only ones, making such a clear-cut physiological distinction unlikely (for a review, see Shapley & Hawken, 2011). Nevertheless, the physiological segregation between pathways at the output of the retina could still lead to differences in the way signals are combined across the eyes.

The spatiotemporal profile of stimuli also appears to impact their binocular combination. For example, matching studies indicate that binocular combination can be close to linear for luminance increments (Anstis & Ho, 1998; Levelt, 1965), particularly against a dark background (Baker, Wallis, Georgeson, & Meese, 2012). This is quite different from the “ocularity invariance” (where binocular and monocular stimuli appear equal) that is well-established when using spatially modulated stimuli, such as sine-wave gratings, and implies strong interocular suppression (Baker & Wade, 2017; Meese, Georgeson, & Baker, 2006; Moradi & Heeger, 2009). Our recent work (Segala et al., 2023) has investigated binocular combination for flickering discs of luminance, which are DC-balanced across time. Steady-state EEG responses from early visual cortex and psychophysical contrast matching data were both consistent with weak interocular suppression when using this stimulus arrangement, but it is currently unclear whether this has implications for contrast discrimination performance.

The main aim of the present study was to characterize binocular signal combination for chromatic stimuli and for temporal modulations of luminance. We also aimed to investigate interocular suppression between chromatic and achromatic pathways. We therefore preregistered a series of psychophysical experiments (see https://osf.io/3vdga/). In Experiment 1, we replicate the four key pedestal masking conditions of Meese, Georgeson, and Baker (2006) described above for achromatic grating stimuli and extend this to both L-M and S-(L+M) isoluminant chromatic stimuli. In Experiment 2, we explore dichoptic masking within and between these stimuli. Experiment 3 repeats the achromatic condition from the first experiment but using a temporally modulated disc rather than sine-wave gratings. We take a Bayesian approach to data analysis and modeling; by fitting a hierarchical version of the two-stage gain control model (Meese, Georgeson, & Baker, 2006), we compare posterior parameter distributions to understand how model parameters such as the weight of interocular suppression vary across visual pathways.

## Materials and methods

### Participants

All experiments were completed by the first author (DHB) and two additional participants, who differed for each experiment. Participants had no known abnormalities of binocular or color vision. Written informed consent was obtained before data collection began, and all procedures were approved by the ethics committee of the Department of Psychology at the University of York (ID number 2202). The study conformed to the tenets of the Declaration of Helsinki.

### Apparatus and stimuli

**Figure 2.**
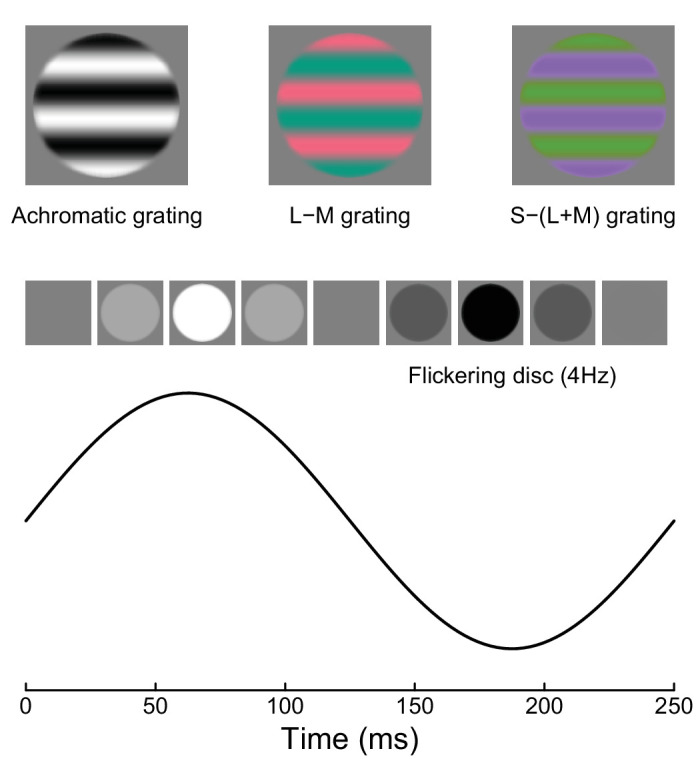
Example stimuli. Upper row shows grating stimuli used in Experiments 1 and 2. Lower row shows one cycle of sinusoidal flicker applied to a uniform disc. Note that the rendering of all stimuli will depend on the device used to display or print this image, and so the chromatic stimuli are unlikely to appear isoluminant, and there may be additional luminance nonlinearities that were not present in the stimuli displayed during the experiments.

In Experiments 1 and 2, the stimuli were horizontal sinusoidal gratings with a spatial frequency of 1 c/deg (see examples in Figure 2). The gratings were windowed by a raised cosine envelope with a diameter of 3 degrees. Spatial phase, relative to a central fixation cross, was randomized on each trial across the four cardinal phases. In the achromatic conditions, the sine-wave modulated all three monitor color channels equally (red, green, and blue). In the L-M condition, we generated isoluminant stimuli for each participant (see Procedures) designed to maximize contrast between L and M cones, while keeping S cone activity constant (more accurately, this is a Δ*L*/*L* − Δ*M*/*M* condition; see Cole, Hine, & McIlhagga, 1993). In the S-(L+M) condition (or, more accurately, the Δ*S*/*S* − (Δ*L*/*L* + Δ*M*/*M*) condition), the isoluminant stimuli maximized S-cone contrast. Stimuli were converted from cone space to monitor RGB coordinates using the monitor spectral readings and the Stockman–Sharpe 2–degree cone fundamentals (Stockman & Sharpe, 2000). The maximum displayable cone contrasts on our system were 0.1 for L-M and 0.88 for S-(L+M). The stimuli in Experiment 3 were temporal modulations of luminance applied to a disc made using the same raised cosine envelope as described above but with no further spatial modulation. The stimuli counterphase flickered sinusoidally at 4 Hz (see examples in the lower portion of Figure 2). In all experiments, we displayed a binocular fusion lock, consisting of three concentric rings of small square elements with random color. A black central fixation cross was also displayed throughout.

All stimuli were presented on an Iiyama VisionMaster Pro 510 CRT monitor, with a refresh rate of 100 Hz and a resolution of 1,024 × 768 pixels. The display was driven by a ViSaGe MkII stimulus generator (Cambridge Research Systems Ltd., Kent, UK) running in 42-bit color mode (14 bits per color channel). We presented stimuli to the left and right eyes independently using a four-mirror stereoscope with front-silvered mirrors. The display was luminance calibrated using a ColorCal photometer (Cambridge Research Systems) and gamma corrected by fitting a four-parameter gamma function to the output of each CRT gun. The maximum luminance was 87 cd/m^2^. We also measured the spectral output of each phosphor using a Jaz spectroradiometer (Ocean Insight, Orlando, FL, USA) and used these measurements to convert between LMS (cone) space and the monitor RGB coordinates. We express threshold and pedestal contrasts in normalized units, relative to the monocular detection threshold for each stimulus type. A threshold value of 2 therefore means that, relative to monocular detection, twice as much contrast was required to reach the same level of performance.

### Procedure

All experiments took place in a darkened room. Participants placed their heads in a chin rest mounted on a height-adjustable table, to which the stereoscope was also attached. The total optical viewing distance (including the light path through the mirrors) was 104 cm, at which distance 1 degree of visual angle encompassed 48 pixels on the monitor.

**Figure 3.**
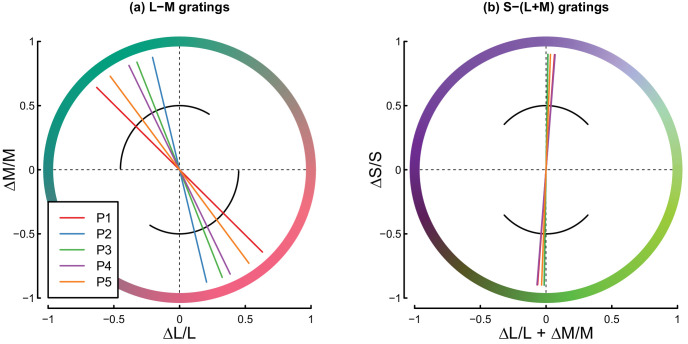
Isoluminance settings from all participants in Experiments 1 and 2. Panel (a) shows L-M and panel (b) shows S-(L+M) settings that were subsequently used to generate stimuli in the main experiments. Within each panel, solid lines show the mean settings for each participant, and black curves show the range of possible stimuli displayed during the adjustment task.

Before beginning primary data collection, each participant in Experiments 1 and 2 completed an isoluminance adjustment task. Grating stimuli were presented that counterphase flickered at 5 Hz, with contrast variations defined about either the L-M or S-(L+M) plane in cone space. Participants used a trackball to dynamically adjust the color angle of the stimulus to minimize the percept of flicker. Each participant completed 10 such trials for each color plane, and the average angle across repetition was taken as the isoluminant point and used to generate stimuli in the main experiment for that participant. Settings were very similar across participants for the S-(L+M) direction and somewhat more heterogeneous for the L-M direction (see Figure 3).

In Experiment 1, participants completed a two-interval-forced-choice (2IFC) contrast discrimination task. Stimuli were presented for 200 ms, with an interstimulus interval of 400 ms. Each interval was indicated by an auditory beep, and participants made their responses using a two-button trackball. Correct responses were followed by a high-pitched tone and incorrect responses by a low-pitched tone. Each block of the experiment tested a single pedestal contrast level and lasted around 12 minutes. The pedestal contrasts were 0%, 0.5%, 1%, 2%, 4%, 8%, 16%, and 32% Michelson contrast for the achromatic stimulus and 0%, 1%, 2%, 4%, 8%, 16%, 32%, and 64% of the maximum available contrast for each chromatic stimulus. On each trial, the target contrast level was determined by a 3-down-1-up staircase procedure, moving in logarithmic (3 dB) steps. There were eight interleaved staircases in total; four stimulus arrangements (see Figure 1) combined factorially with two target eye assignments. Each pedestal contrast was repeated three times by each participant, and the block order was randomized. The experiment lasted around 4 hours per participant for each chromatic condition and took place over the course of several weeks. In total, the experiment consisted of 67,978 trials (pooled across participants).

In Experiment 2, participants completed a 2IFC dichoptic masking task. The stimuli and trial protocol were the same as for Experiment 1, except that the target contrast was chosen from a set of 10 possible values, determined in advance based on the data of Experiment 1. There were 12 possible conditions: baseline detection thresholds for achromatic, L-M, and S-(L+M) stimuli and the nine possible factorial pairings obtained by assigning these conditions to be target and dichoptic mask stimuli. Mask contrasts were chosen to be approximately 16 times their (monocular) detection threshold, based on the data from Experiment 1. The contrasts were 16% Michelson contrast for the achromatic stimuli, 6.4% cone contrast for L-M, and 56% cone contrast for S-(L+M). Each block of the experiment tested a single condition and consisted of 200 trials. A high-contrast example of the target stimulus was displayed at the foot of the screen throughout, so that there was no ambiguity about the target identity on a given block. Participants completed 10 repetitions of each condition (120 blocks of ~6 minutes each), lasting around 12 hours, for a total of 72,000 trials (pooled across participants).

In Experiment 3, the achromatic conditions from Experiment 1 were repeated using a flickering disc stimulus. The stimulus counterphase flickered at 4 Hz and was presented for 500 ms (i.e., two full cycles of the temporal modulation). All other procedures were the same as for Experiment 1, and the experiment comprised a total of 24,610 trials (pooled across participants).

### Data analysis and computational modeling

Psychometric functions from each experiment were fit using *psignifit* 4 to estimate threshold and slope parameters via a Bayesian numerical integration method (Schütt, Harmeling, Macke, & Wichmann, 2016). A cumulative Gaussian was used as the underlying function, with contrast values expressed in decibel (dB) units (where $C_{dB}=20\log_{10}\left(C_{\%}\right)$). We converted the slope estimates (σ parameters from the fitted Gaussians) to equivalent Weibull β values using the approximation β = 10.3/σ. Threshold was defined as the target contrast corresponding to an accuracy of 75% correct.

The two-stage model of Meese, Georgeson, and Baker (2006) was fit to the threshold data from Experiments 1 and 3 using a simplex algorithm to minimize the error between the model and data. We normalized the thresholds and pedestal contrasts to the appropriate monocular threshold for each stimulus type. The model is defined by a series of equations:

(1) $$\mathrm{Stage}1_{L}=\frac{C_{L}^{m}}{S+C_{L}+\omega C_{R}},$$

(2) $$\mathrm{Stage}1_{R}=\frac{C_{R}^{m}}{S+C_{R}+\omega C_{L}},$$

(3) $$\mathrm{binsum}=\mathrm{Stage}1_{L}+\mathrm{Stage}1_{R},$$

(4) $$\mathrm{Stage}2=\frac{{\mathrm{binsum}}^{p}}{Z+{\mathrm{binsum}}^{q}},$$

where *C*_*L*_ and *C*_*R*_ are the (normalized) contrasts displayed to the left and right eyes, and *m*, *S*, ω, *p*, *q*, and *Z* are free parameters in the model. A further free parameter, *k*, is used to convert the model outputs to either d-prime or threshold values (note that in the original model specification [Meese, Georgeson, & Baker, 2006], this parameter was called σ, but we use the *k* symbol here to avoid confusion with the standard deviation of the cumulative Gaussian used when fitting the psychometric functions to estimate thresholds). Thresholds are defined by iteratively adjusting the target contrast until the following equality is satisfied:

(5) $$\mathrm{Stage}2_{\mathrm{target}+\mathrm{pedestal}}-\mathrm{Stage}2_{\mathrm{pedestal}}=k,$$

and *d*′ (d-prime) for a single target level is defined as

(6) $$d^{'}=\frac{\mathrm{Stage}2_{\mathrm{target}+\mathrm{pedestal}}-\mathrm{Stage}2_{\mathrm{pedestal}}}{k/\tau },$$

where the parameter τ reflects the value of *d*′ at detection threshold. Defining threshold as the 75% correct point on the psychometric function (as here) yields a value of $\tau =\Phi^{-1}\left(0.75\right)\sqrt{2}$ = 0.954 (where Φ^−1^ is the inverse cumulative normal density function). The combined denominator term (*k*/τ) therefore represents internal additive noise in the model.

We ran the simplex algorithm from 100 random starting vectors for each data set and chose the solution for each data set that gave the smallest root mean squared error (RMSE) between the model and data.

We also implemented a Bayesian hierarchical version of the model using the Stan probabilistic programming language (Carpenter et al., 2017). This used a binomial generator function to model the proportion correct data at each target level and was fit simultaneously to all participants for a given experiment, but separately for each chromatic condition of Experiment 1, and the flickering disc data from Experiment 3 (i.e., four fits in total, as for the simplex fitting). Prior distributions for the parameters *p*, *q*, *m*, and ω were Gaussian, with means determined from published values (see first row of Table 1). Priors for parameters *S*, *Z*, and *k* were uniform. This modeling primarily focuses on examining posterior parameter distributions, rather than a model comparison approach. We generated over 1 million posterior samples for the model and retained 10% of them for plotting.

**Table 1.** Summary of fitted model parameters. The top row gives the best-fitting parameters from the study of Meese, Georgeson, and Baker (2006). The second section shows the best-fitting parameters from simplex fits to the averaged thresholds for each experiment. The final rows show the posterior parameter estimates from the Bayesian model, fitted to each data set from Experiments 1 and 3.

Model fit	*p*	*q*	*m*	*S*	*Z*	*ω*	*k*	RMSE
Meese, Georgeson, and Baker (2006)	7.99	6.59	1.28	0.99	0.08	1.00	0.19	
Simplex fits								
Achromatic gratings	5.96	4.69	1.34	0.87	0.13	1.00	0.20	1.59 dB
L-M gratings	7.77	5.19	1.17	0.55	0.07	1.00	0.17	1.39 dB
S-(L+M) gratings	16.01	11.56	1.13	0.33	0.00	0.99	0.27	1.46 dB
Flickering discs	5.62	4.51	1.26	0.95	0.25	0.89	0.13	1.05 dB
Bayesian model								
Achromatic gratings	7.06	5.63	1.27	0.63	0.08	0.98	0.30	
L-M gratings	5.11	3.63	1.24	1.22	0.03	1.05	0.19	
S-(L+M) gratings	6.78	5.34	1.24	0.61	0.01	0.95	0.34	
Flickering discs	7.16	5.76	1.25	0.65	0.13	0.87	0.24	

Finally, we adapted the two-stage model to include parallel pathways to process achromatic, L-M, and S-(L+M) stimuli that mutually suppress each other. We added additional suppressive terms at the first (monocular) stage of the model, for example:

(7) $$\begin{array}{c} AC\mathrm{Stage}1_{L}=\frac{AC_{L}^{m}}{S+AC_{L}+\omega_{A}AC_{R}+\omega_{R}RG_{R}+\omega_{B}BY_{R}},\; \end{array}$$

where *AC* represents the achromatic contrast, *RG* represents the L-M contrast, *BY* represents the S-(L+M) contrast, and ω_*A*_, ω_*R*_, and ω_*B*_ are the accompanying weights of interocular suppression. There is an equivalent expression for the right eye and for each of the two isoluminant chromatic pathways. To simplify the model and avoid free parameters that are poorly constrained by the data, we fixed several parameters (*p*, *q*, *m*, *S*, *k*, and ω for the within-pathway suppression) at the values from the fits from Experiment 1 (Table 1, lower rows). This left nine free parameters: a *Z* parameter for each mechanism and six cross-mechanism weights of interocular suppression. These parameters were again estimated within a Bayesian hierarchical framework, using the binomial proportion correct data from Experiment 2. Note that the model as specified does not currently include monocular suppression between different pathways, as we did not collect any data for these conditions. Previous work (e.g., Chen et al., 2000; Mullen & Losada, 1994) has measured such interactions, and they could in principle be incorporated into the denominator of either Stage 1 or Stage 2 in the model.

### Open science practices

All experimental code, raw data, and analysis scripts are available at https://osf.io/3vdga/. The linked GitHub repository also contains a fully computationally reproducible version of the manuscript. Note that we deviated slightly from the planned preregistration, in that we did not collect data for chromatic flickering discs or for the cross-pathway dichoptic experiment using disc stimuli. This is because the grating data from Experiments 1 and 2 and the achromatic disc data from Experiment 3 were sufficient to address the questions we had hoped to answer from these experiments.

## Results

### Experiment 1

**Figure 4.**
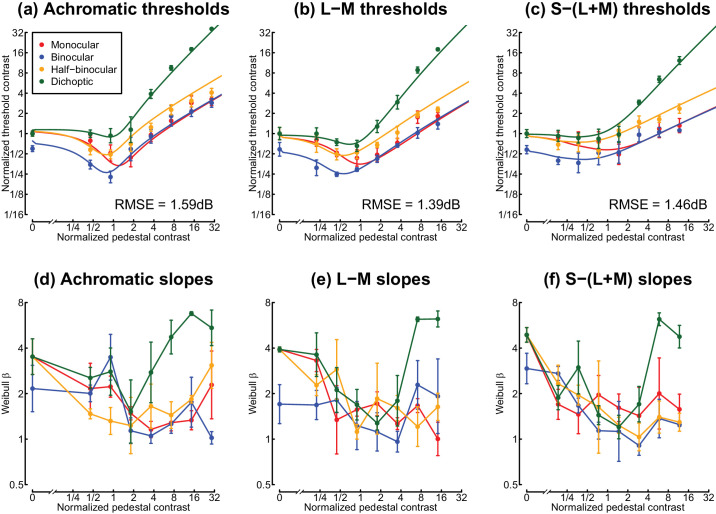
Dipper functions and psychometric slopes from Experiment 1, averaged across three participants. Panels (a–c) show threshold data, and panels (d–f) represent the slope of the psychometric function expressed in Weibull β units. Error bars give ±1SE across participants. Note that all contrast values for both thresholds and pedestals are normalized to the monocular detection threshold in each panel. Curves in panels (a–c) show the best-fitting models, optimized using a simplex algorithm, and RMSE values give the root mean squared errors of the fits.

Dipper functions from Experiment 1 are displayed in the upper row of Figure 4. Panel (a) shows the achromatic results, which replicate the key features from previous work. At detection threshold, binocular summation was a factor of 1.67 (4.47 dB), within the range ($\sqrt{2}$ to 2) consistent with previous reports (Baker et al., 2018). Pedestal masking functions followed the typical “dipper” shape in all conditions, with a region of facilitation at low pedestal contrasts and masking at higher contrasts. The monocular and binocular dipper handles converge at high contrasts, whereas the half-binocular thresholds remain above the binocular thresholds across the full range of pedestal contrasts. The dichoptic condition produced very high thresholds, with the rising portion of the dipper having a slope around 1 (regression slope of 1.06 in log (dB) units, calculated across the highest four pedestal contrasts).

**Figure A1.**
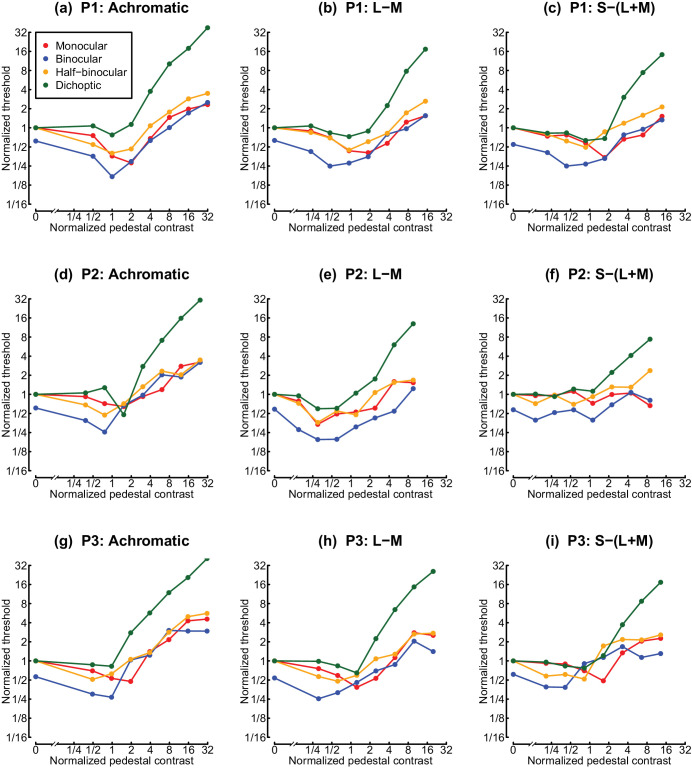
Individual participant data from Experiment 1. Panels (a, d, g) show achromatic data, panels (b, e, h) show L-M data, and panels (c, f, i) show S-(L+M) data.

A similar pattern of results was observed for both the L-M and S-(L+M) isoluminant stimuli (see Figures 4b, c). Summation at threshold was a factor of 1.71 (4.66 dB) for the L-M targets and a factor of 1.73 (4.77 dB) for the S-(L+M) targets, and so was marginally higher than for achromatic stimuli. The general character of the dipper functions was largely consistent with the achromatic results, though we observed shallower facilitation and weaker masking, especially for the S-(L+M) stimuli. For example, the strongest facilitation in the binocular condition for achromatic stimuli was a factor of 2.63, whereas it reduced to a factor of 2.35 for L-M stimuli and 1.57 for S-(L+M) stimuli. The slope of the binocular dipper handle was 0.52 for achromatic stimuli, 0.58 for L-M stimuli, and 0.34 for S-(L+M) stimuli. Dichoptic masking remained as strong for the chromatic conditions as for the achromatic stimuli (regression slopes of 1.3 for L-M and 1.21 for S-(L+M)). The pattern of results for individual participants was consistent with the group averages, as shown in Figure A1.

**Figure 5.**
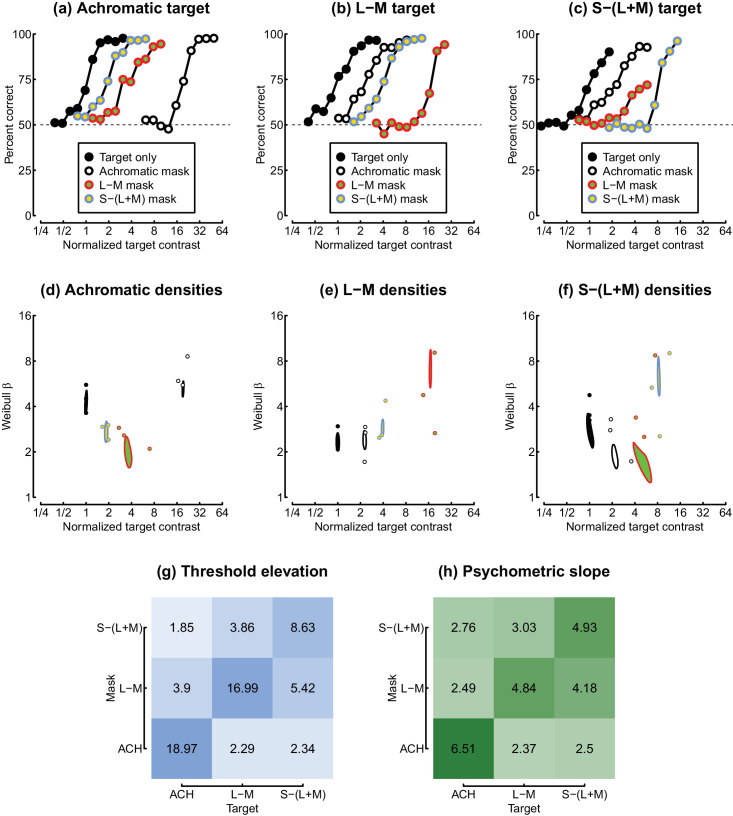
Summary of data from Experiment 2. Panels (a–c) show psychometric functions for each condition, pooled across participants (600 trials per target contrast level). Panels (d–f) show threshold and slope estimates for individual participants (points) and the boundary of the posterior density estimates for fits to the pooled data (ellipses). Panel (g) shows the average threshold elevation factor for each combination of target and mask stimulus. Panel (h) shows the geometric mean psychometric slope value for each masking condition, expressed in Weibull β units. Contrast values are normalized to the target-only threshold for each stimulus type.

**Figure 6.**
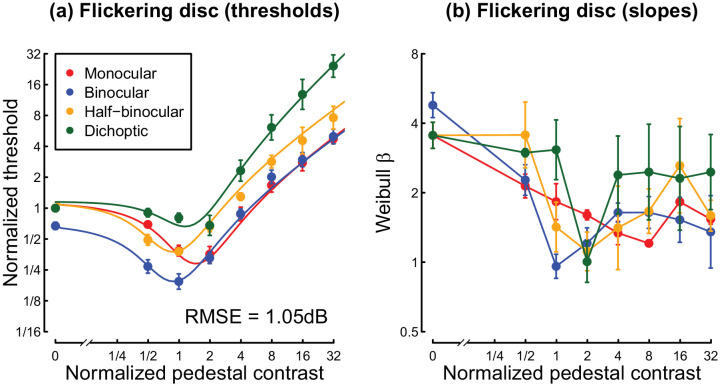
Thresholds (a) and psychometric slopes (b) for the flickering disc experiment. Plotting conventions mirror those in Figure 4.

**Figure 7.**
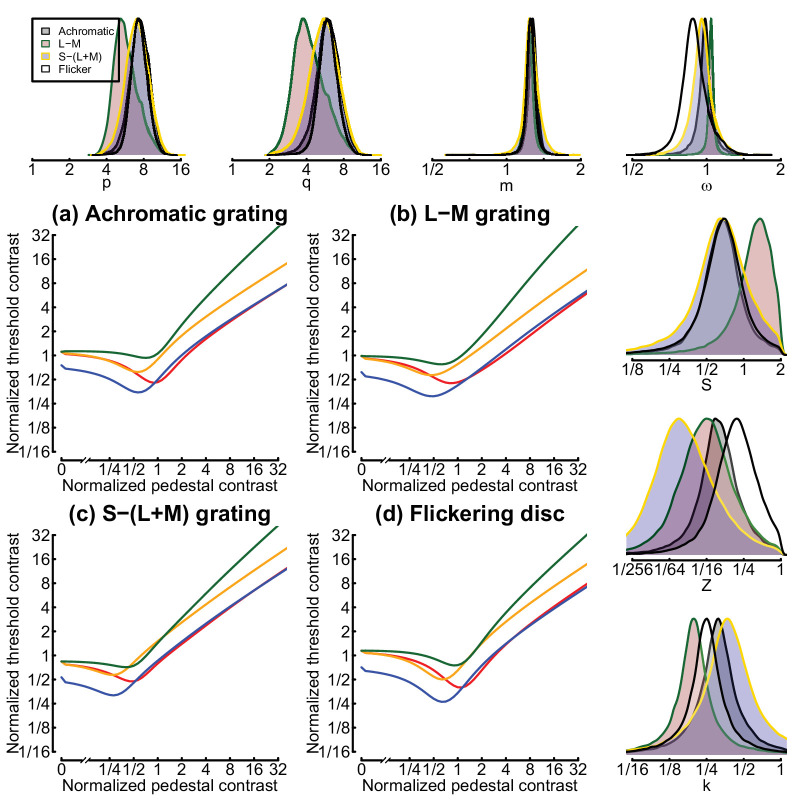
Model predictions (a–d) and posterior parameters (top and right margin plots) for the hierarchical Bayesian model. Curves in panels (a–d) show thresholds generated using the maximum a posteriori parameter estimates. The probability density functions in the margin plots are peak-normalized and shown for each of the four data sets in different colors (see legend in upper left plot). Distributions were generated from 1,000,000 samples per data set, using a Markov chain Monte Carlo sampling algorithm. Note the logarithmic x-axis for all posterior plots.

**Figure 8.**
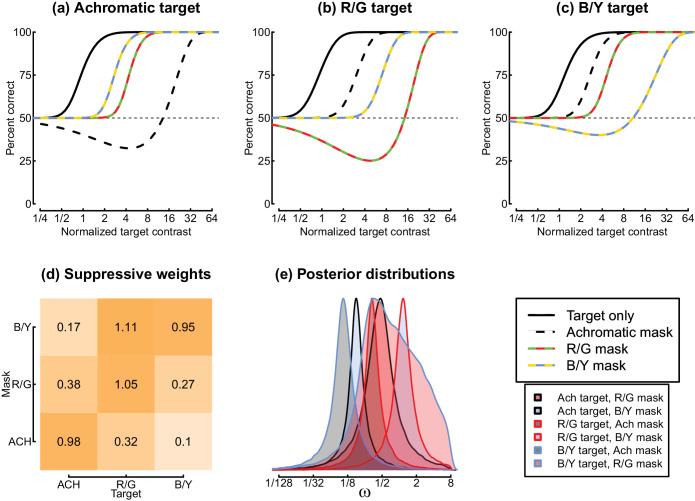
Summary of the model fit to the data of Experiment 2. Panels (a–c) show model psychometric functions in the same format as those in Figure 5. Panel (d) shows the fitted suppressive weights (ω values) estimated by the model, and panel (e) shows the posterior distributions for each weight parameter. The lower right plot gives the legends for the upper row (top) and the posterior distributions in panel (e).

Following Meese, Georgeson, and Baker (2006), we also inspected the slope of the psychometric function for each condition (see Figures 4d–f), as this provides information about the effective gradient of the underlying contrast response function. At detection threshold, slopes were relatively steep for all stimuli, with values around β = 4. As pedestal contrasts increased, slopes linearized and reduced to around β = 1.3 (Foley & Legge, 1981; Meese, Georgeson, & Baker, 2006) and remained shallow at high pedestal contrasts. The exception to this was the dichoptic condition, where slopes became extremely steep at high dichoptic mask contrasts, consistent with previous observations (Baker, Meese, & Georgeson, 2013; Meese, Georgeson, & Baker, 2006). This was clear for all three data sets, with slope values in the range 4 < β < 8. However, we observe that slope estimates are more variable than threshold estimates (note the large error bars), particularly when using adaptive staircases, which deploy most trials close to threshold. Our second experiment therefore investigated the slope of the psychometric function in more detail for dichoptic masking using the method of constant stimuli, as well as exploring dichoptic interactions between chromatic channels.

### Experiment 2

**Figure A2.**
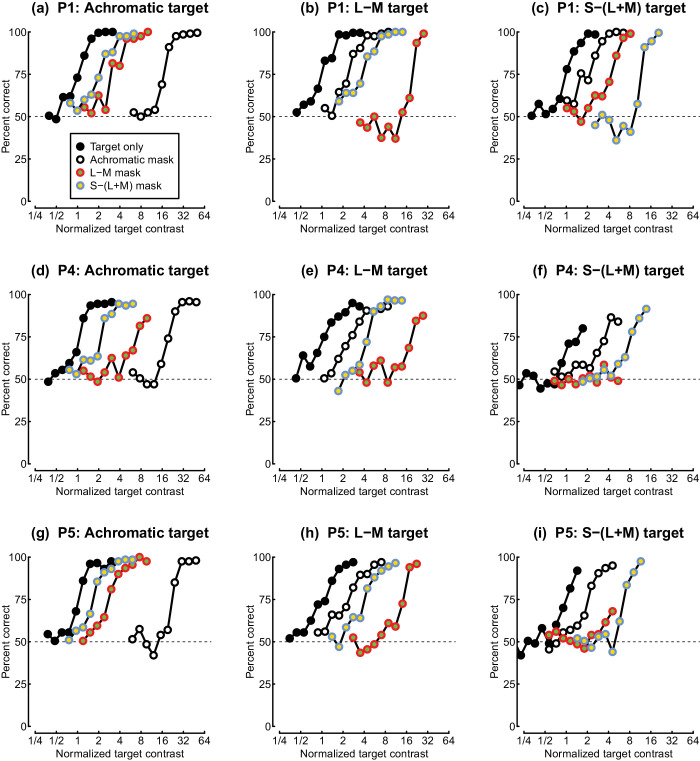
Individual participant data from Experiment 2. Panels (a, d, g) show achromatic data, panels (b, e, h) show L-M data, and panels (c, f, i) show S-(L+M) data.

In Experiment 2, we focused on the dichoptic condition at a single mask contrast and measured full psychometric functions using the method of constant stimuli for all factorial pairings of target and mask chromaticity. The pooled results across three participants are shown in Figures 5a–c, and results for individual participants are available in Figure A2. All conditions produced monotonically increasing psychometric functions (panels a–c), but the extent of masking was highly dependent on the relationship between the target and mask chromaticity. Figures 5d–f show a two-dimensional representation of individual threshold and slope estimates (points), as well as the posterior density estimates for fits to the pooled data (ellipses). The results are consistent between participants and, at the group level, show that the presence of a mask has a strong effect on thresholds.

Threshold elevation was greatest when the target and mask had the same chromaticity—notice that the psychometric function is shifted furthest to the right for the achromatic target with an achromatic mask (white and black circles in Figure 5a), for the L-M target with an L-M mask (red and green circles in Figure 5b), and for the S-(L+M) target with a S-(L+M) mask (blue and yellow circles in Figure 5c). Masking was weakest between achromatic masks/targets and chromatic masks/targets. Finally, there was an intermediate level of masking between L-M and S-(L+M) stimuli. This is summarized in Figure 5g, which represents threshold elevation for each combination of target and mask chromaticity. Note that the positive diagonal exhibits the highest values and represents threshold elevation between targets and masks of the same chromaticity.

We also calculated the slope of the psychometric function for each condition, in equivalent Weibull β units. In the absence of a mask, the average slope was β = 3.42, which is typical for contrast detection tasks (Wallis, Baker, Meese, & Georgeson, 2013). Slopes became substantially steeper when the dichoptic mask matched the target in chromaticity (average β = 5.38). These “super-steep” psychometric functions for dichoptic pedestal masking have been reported previously (Baker et al., 2013; Meese, Georgeson, & Baker, 2006) and are observed for the first time here using chromatic stimuli (see diagonal values in Figure 5h and also Figures 4d–f). However, we did not see such markedly steep functions for any of the cross-chromaticity masking conditions (average β = 2.83 for the off-diagonal values).

### Experiment 3

**Figure A3.**
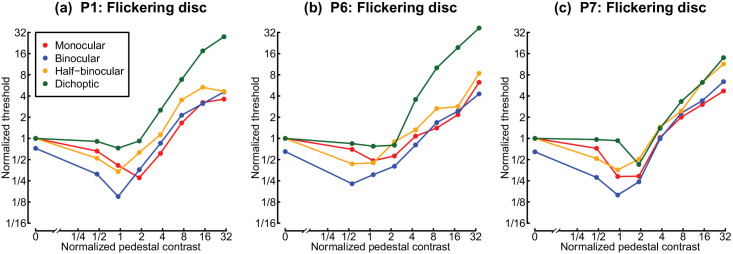
Individual participant data from Experiment 3. Each panel shows a different participant (a: P1, b: P6, c: P7).

In our final experiment, we again measured dipper functions, but this time for a disc temporally modulating in luminance. This was motivated by our recent work (Segala et al., 2023) that appeared to show increased binocular facilitation and reduced interocular suppression for flickering disc stimuli (relative to gratings), measured using EEG and a psychophysical matching paradigm. The pattern of dipper functions for a 4 Hz flickering disc (see Figure 6a) was very similar to that observed for achromatic gratings (see Figure 4a), and the binocular summation ratio at threshold was also similar (a factor of 1.49 for discs vs. 1.67 for gratings). Threshold data for individual participants are shown in Figure A3. We also found a similar pattern of psychometric slope values (Figure 6b) as we had for gratings, though we note that the dichoptic condition did not produce the “super-steep” psychometric functions we had observed in Experiments 1 and 2. Nevertheless, the dichoptic slopes are somewhat above those of the other pedestal arrangements at high contrasts.

### Computational modeling

For consistency with previous work, we initially performed least squares fits of the two-stage model (Meese, Georgeson, & Baker, 2006) for each of the chromaticity experiments and the flickering disc experiment (seven free parameters per fit). The best model fits are shown by the curves in Figures 4 and 6 and provide an excellent description of the data, with RMS errors between 1.05 and 1.59 dB. Best-fitting parameters are shown in the “Simplex fits” section of Table 1. We note that in previous work, the weight of interocular suppression (ω in the model) was implicitly fixed at 1. Here we allowed it to vary, but it still received a value very close to 1 in all of our grating conditions and slightly below 1 (0.89) for our flickering discs. The exponent values (*p* and *q*) for the S-(L+M) gratings are rather different from those of the other conditions. In previous work (Meese, Georgeson, & Baker, 2006), the second stage gain control nonlinearity (Equation 4) does typically have quite substantial exponent values, which balance the relatively mild nonlinearity at the first stage and produce a compressive transducer that results in contrast masking (the handle of the dipper). The high value of *p* = 16.01 is therefore likely to be compensating for the low value of *m* = 1.13 at Stage 1 but may well represent the combination of several successive stages of nonlinearity and perhaps also other phenomena such as uncertainty (Pelli, 1985).

We additionally implemented a hierarchical Bayesian version of the model to estimate full posterior parameter distributions. This model was fit simultaneously to the full trial-by-trial data from all participants who completed a given experiment (i.e., the model was fitted separately to each of the four dipper data sets). Figures 7a–d summarize the model behavior, which displays the same pattern of dipper functions as we found empirically. Modal posterior parameter values (maximum a posteriori [MAP] estimates) are given in the lower rows of Table 1. These are in a similar range to the parameters from the simplex fitting and the original (Meese, Georgeson, & Baker, 2006) parameters (reproduced in the first row of the table for reference). The panels along the right and upper margins of Figure 7 show posterior distributions for each model parameter. Note in particular that the posterior distribution for the weight of interocular suppression (ω, top right panel) overlaps 1 for each experiment, consistent with the strong dichoptic masking observed in the threshold data. We note that the distribution for ω in the flickering disc condition is somewhat lower than for the other three data sets. However, this difference is not meaningful according to widely accepted criteria such as comparing the 95% intervals of the distribution to a value of ω = 1.

Finally, we fitted an extended model that included interocular suppression between the different pathways (see Equation 7) to the data of Experiment 2. Figure 8 shows the model curves (panels a–c), which correspond closely to the data in Figure 5. One striking discrepancy is that the model predicts a region of negative d-prime for the case where the target and dichoptic mask have the same chromaticity (see the curved regions below 50% correct). This happens in the model because low-contrast targets suppress the mask more than they excite the detecting mechanism, producing a net decrease in response. The feature (termed a “swan function”) was not generally present in our empirical data, though it can be observed for one participant in Figures A2b, c. Our previous work (Baker et al., 2013) has found evidence for this phenomenon, but it generally requires very high mask contrasts to be measurable empirically, and there may also be some individual differences; both factors might explain its absence here.

Figures 8d, e show the model estimates for the weight of interocular suppression in each combination of target and mask chromaticity. The weakest suppression was between achromatic targets and masks and S-(L+M) targets and masks, and the strongest suppression was between L-M targets and S-(L+M) masks. Note that the weight parameter value of ω = 1.11 for this condition is slightly higher than that of the within-channel weights (values around 1), even though the within-channel conditions generate more threshold elevation. In the model, high thresholds for within-channel dichoptic masks occur because the target and mask are summed together, which causes additional masking (see Baker et al., 2013). However, it is clear that the posterior distribution of the weight parameter is quite broad and overlaps 1, so this difference may not be meaningful. There do not appear to be any other salient patterns in the suppressive weight estimates.

## Discussion

Across three psychophysical experiments, we have demonstrated that:

• Binocular combination of isoluminant chromatic stimuli is similar to that for achromatic stimuli.
• Interocular suppression is strongest within a postretinal pathway and weakest between the achromatic and S-(L+M) pathways.
• Binocular combination occurs similarly for spatial and temporal luminance modulations.

We now discuss the relationship to previous work and consider the likely physiological substrates of these effects.

### Summation at threshold

Estimates of the binocular summation ratio at threshold fell within the range $\sqrt{2}$ to 2 for all four stimuli tested here. Consistent with previous reports (Simmons, 2005), summation was slightly higher for chromatic versus achromatic stimuli but fell short of perfect linear summation (a ratio of 2). In our model, summation at threshold is determined by the exponent at the first gain control stage (the *m* parameter in Equation 1 and Equation 2). The summation ratio can be approximated by 2^1/*m*^ (see figure 9 of Baker et al., 2012), such that an exponent of *m* = 1 produces a ratio of 2, and an exponent of *m* = 2 produces a ratio of $\sqrt{2}$. Both the simplex and Bayesian model fits (see Table 1) generated parameter estimates in the range 1 < *m* < 1.4, consistent with the high levels of summation observed empirically. Note that the exponents at Stage 2 (*p* and *q*) have no effect on binocular summation at threshold, because they impact after the signals have been combined. The differences in these parameter values between chromatic conditions (see Table 1) primarily reflect differences in the slope of the dipper handle (chromatic conditions are shallower) and are of incidental interest for our main questions.

### Interocular suppression

The weight of interocular suppression is represented by the model parameter, ω (Equation 1 and Equation 2). Posterior distributions of this parameter overlapped 1 for all of our dipper function data sets (see top right panel in Figure 7), indicating strong interocular suppression regardless of postretinal pathway. The flickering disc stimuli produced a slightly lower suppression estimate, with ω < 0.9. This may indicate slightly weaker suppression between the eyes for temporal modulations, but it is much less extreme than our recent estimates using EEG and matching paradigms (Segala et al., 2023). One difference between these studies is the luminance of the background, which was set to black for the experiments of Segala et al. (2023), but here was set at the mean luminance. Other studies using static luminance increments have also reported differences in the character of binocular combination that are attributable to background luminance (Baker et al., 2012), so this may explain the differences between studies. We also note that the weaker interocular suppression for flickering disc stimuli appears to be largely due to one participant (see Figure A3c), so individual differences might also play a role. Future work should manipulate the background luminance systematically to better understand how this modulates interocular suppression.

Strong interocular suppression is responsible for the convergence of monocular and binocular dipper handles (e.g., Legge, 1984) at high pedestal contrasts (see Figure 4). In the model, monocular stimuli avoid the impact of interocular suppression, because the unstimulated eye sees a blank screen and so contributes a contrast of zero to the denominator of the stimulated eye. Binocular stimuli receive strong suppression, which acts to approximately halve the excitation when contrasts in the two eyes are equal (see Georgeson et al., 2016; Meese, Georgeson, & Baker, 2006). This normalization leads to very similar responses for monocular and binocular stimulation. In the auditory system, binaural combination of amplitude modulated signals involves much weaker suppression between channels, and the monaural and binaural dipper handles do not converge at high contrasts (Baker et al., 2020).

One phenomenon that often occurs with conflicting dichoptic stimuli is binocular rivalry, and it is worth considering how this might impact the results of Experiment 2, where the two eyes often saw stimuli of different chromaticities. However, rivalry typically only “kicks in” at presentation durations longer than the 200 ms used here (see, e.g., Wolfe, 1983), and certainly rivalry alternations need much longer presentations to be clearly observed. In a dichoptic masking paradigm, it is often difficult to distinguish dichoptic fusion from dominance of one eye over the other; indeed, interocular suppression of sufficient strength would render the weaker stimulus invisible. On the other hand, if the stimuli appear fused, we might expect dichoptic color mixing to occur (Kingdom & Libenson, 2015; Kingdom & Wang, 2015), whereas if one eye dominates, the color percept would be equivalent to monocular presentation of the dominant stimulus. Here we used a performance task (2AFC detection/discrimination) and did not explicitly ask participants about the stimulus appearance. Future studies could use matching and adjustment tasks to investigate this further.

### Psychometric slopes

Previous work has demonstrated that the slope of the psychometric function in 2AFC tasks can distinguish different types of masking, although it is much less widely reported than threshold measures. In particular, pedestal masking linearizes the slope (Foley, & Legge, 1981; Meese, Georgeson, & Baker, 2006), and within-channel dichoptic masking produces very steep slopes (Baker et al., 2013; Meese, Georgeson, & Baker, 2006). We replicate both of these effects here and show that they extend to the isoluminant chromatic pathways (see Figures 4d–f and 5). We additionally show that dichoptic masking between different pathways does not produce unusually steep slopes (see Figure 5). It therefore more closely resembles other types of masking between visual channels, such as cross-orientation masking (Meese & Baker, 2009), surround masking (Yu, Klein, & Levi, 2002), and masking from broadband noise (Baker & Meese, 2012; Lu & Dosher, 2008), which also do not impact psychometric slopes.

### Physiological substrates

Recent evidence indicates that the physiological substrate of interocular suppression may be neurons in Layer 4 of primary visual cortex (Dougherty, Cox, Westerberg, & Maier, 2019). Most cells in this layer are monocularly excitable, in that their responses increase only by stimulation of their preferred eye. However, simultaneous stimulation of the nonpreferred eye can modulate the response, usually in an inhibitory fashion, exactly as proposed at Stage 1 of the two-stage model (Equation 1 and Equation 2). In terms of perception, one consequence of this early suppression is to achieve “ocularity invariance,” whereby the perceived contrast of a stimulus viewed by one eye is equivalent to that of the same stimulus viewed by both eyes (Baker, Meese, & Georgeson, 2007). Similar processes of response invariance have also been reported using fMRI (Moradi & Heeger, 2009) and steady-state EEG (Baker & Wade, 2017).

In V1, the classical view was that chromatic stimuli are processed in “blob” regions that are largely monocular as they fall within ocular dominance columns (Livingstone & Hubel, 1984). However, more recent work has shown chromatic processing outside of the blobs (Sincich & Horton, 2005), especially for stimuli with spatial structure (Chatterjee, Ohki, & Reid, 2021). Since our psychophysical results indicate that interocular suppression is equally strong within chromatic and achromatic pathways, it may be that psychophysical performance indexes a common population of (nonblob) neurons for all of our stimuli. On the other hand, if blob regions include a subset of monocular neurons subject to interocular suppression (e.g., Dougherty et al., 2019), this might be followed by binocular summation at a later stage of processing for chromatic stimuli and potentially also for very low spatial frequency luminance modulations also processed in blobs (Economides, Sincich, Adams, & Horton, 2011).

## Conclusions

Here we provide estimates of interocular suppression within and between the three primary postretinal visual pathways. These results show that binocular signal combination is similar within each pathway but that interocular suppression is typically weaker between pathways. Our findings could be applied when building models to predict perception of binocular images and movies, for example, those generated by virtual and augmented reality systems, or in three-dimensional cinema and television.
